# Understanding the Use of Community Involvement in Rural Food Environment Modifications: A Systematic Review

**DOI:** 10.1007/s13668-025-00674-9

**Published:** 2025-06-28

**Authors:** Yin Liew, Leanne J. Brown, Renaye Madden, Laura Alston, Lisa Urquhart, Susan Heaney, Tracy Schumacher

**Affiliations:** 1 School of Health Sciences, University of Newcastle, Callaghan, New South Wales, Australia; 2 Department of Rural Health, University of Newcastle, Tamworth, New South Wales, Australia; 3 Department of Rural Health, University of Newcastle, Moree, New South Wales Australia; 4 Department of Rural Health, Deakin University, Colac, Victoria Australia; 5 Department of Rural Health, University of Newcastle, Coffs Harbour, New South Wales Australia; 6 Department of Rural Health, University of Newcastle, Port Macquarie, New South Wales Australia

**Keywords:** Food environment, Nutrition, Health, Rural, Community

## Abstract

**Purpose of Review:**

Rural communities experience higher rates of adverse health outcomes compared to their urban counterparts, and involving community members in intervention design and implementation is increasingly recognized as a pivotal factor in fostering successful intervention outcomes. This review aims to synthesize the evidence on rural food environment modifications globally, and to understand the role and involvement of community members in these interventions. Eligible records were dated between 1st January 2011 and 6th September 2024. Two independent reviewers conducted screening, quality assessment, and data extraction. Studies were included if they addressed a food environment, described food environment modifications, indicated community involvement, were based in regional or rural areas and published in English. The Mixed Methods Appraisal Tool was used to assess the risk of bias. Results were reported as a narrative synthesis.

**Recent Findings:**

Thirty-three studies were eligible for inclusion following appraisal of full texts, comprised of 46 reports. Of these, 12 studies (16 reports) focused on First Nations populations and will be reported separately, due to the complex and sensitive nature of engaging with these communities. Therefore, 21 studies (30 reports) were included in this review. Study designs were heterogenous. Restaurants, supermarkets and schools were a common focus for modifications, while some programs simultaneously targeted several food environments. However, community involvement was inconsistently reported across studies, making conclusions about involvement difficult.

**Summary:**

Standardized reporting of the involvement of community members is needed to ensure systematic and authentic approaches to community involvement are integrated into rural public health interventions and research to enhance success.

**Registration:**

Prospero; CRD42023400455.

**Supplementary Information:**

The online version contains supplementary material available at 10.1007/s13668-025-00674-9.

## Introduction

The food environment relates to factors that influence an individual’s day-to-day access to food. It consists of all venues where people procure and consume food, such as supermarkets, small retail stores, cafes, school canteens, and restaurants [1]. The food environment has been conceptualized by Turner et al. as being comprised of an external domain related to production and sale of foods and a personal domain related to acquisition and consumption [2]. The external domain includes factors such as food availability, prices, product attributes, marketing, and regulation, while the personal domain consists of individual-level factors including food accessibility, affordability, convenience, and desirability [2]. These domains are interconnected and can have a considerable impact on population nutrition-related outcomes. When supportive policies and interventions are in place, these can positively influence consumer choice during the procurement of food items [3]. 

Food intake is influenced by a variety of factors (e.g. individual beliefs, family influence, policies and regulations) of which choice is a key component [4]. Food choice is a complex process that is impacted by individual factors and the local food environment [2, 5, 6]. Access to nutritious and affordable foods is an issue that is more evident for those located in rural and remote areas compared to urban areas [7]. Individuals in rural or remote communities encounter difficulties when purchasing affordable and nutritious foods primarily due to geographical isolation. Long haul transportation impacts transport costs and poses additional challenges for the logistics related to delivery of fresh or perishable food items to designated remote locations [8]. 

Emerging evidence suggests that the key to improving nutrition-related outcomes is to intervene in the food environment and ensure nutritious, safe, and affordable food is available and accessible to all within the community regardless of location [5, 9, 10]. However, all communities are unique and include variations in food consumption patterns, customs, traditions, and practices across different population groups [11, 12]. In order to have successful food environment modifications in rural areas, it is important to engage with those living in rural communities to help overcome the barriers they experience in their local food environment [13, 14]. 

Community involvement or community engagement in this instance is defined as the process of working collaboratively with groups of individuals associated with geographic proximity. These groups usually share interests, or comparable circumstances concerning issues affecting their well-being [13]. The involvement of the community encompasses the local experiences, cultural values and stakeholders’ perspectives which allow interventions to take place successfully, ensure the sustainability of interventions in the long run and result in better outcomes [6, 13, 14]. While the involvement of the community in intervention planning and implementation has been widely documented in nutrition interventions and other health or non-health studies [15–17], the extent of community involvement in rural food environment modifications has not been examined. A 2022 systematic review suggested the benefits of co-creation in food environment modifications (e.g. growing partnerships within communities and sectors, empowerment of community and retailers, cultural appropriateness) however, it focuses on food retail environments with no specific mention of rurality [16]. A 2020 review demonstrated the effectiveness of rural retail environment modifications in supporting the procurement of healthier options in stores, however, the lack of community involvement in the studies was not defined [17]. Therefore, this review aims to synthesize the evidence on rural food environment modifications globally, and to understand the use of community in these interventions. Specifically, this review is focused on the following research questions:

i. What community involvement was there in rural food environment modifications?
ii. How did community involvement influence the modifications to the rural food environment?
iii. Which part of the food environment did the modification aim to address?
iv. What modifications were made to rural food environments?

## Methods

This systematic review was registered with PROSPERO International prospective register of systematic reviews (ID: CRD42023400455; Date: 27 February 2023), and reported in accordance with the Preferred Reporting Items for Systematic Reviews and Meta-Analyses (PRISMA) 2020 guidelines [18]. 

### Eligibility Criteria

The food environment modification/s needed to be performed in a population designated by the authors of the paper as living in a rural, regional, remote, non-urban or non-metropolitan area. No set definition of ‘rurality’ was assumed, as although an international definition exists, it is acknowledged that ‘rurality’ is attributed different meanings or understanding among different countries and communities [19, 20]. Community involvement was broadly defined as any stated involvement from members belonging to the geographic community, and may encompass retailers, consumers, stakeholders, reference groups or any other community members relevant to the modification. Modification to the food environment could include any program, intervention, or activity that was related to the healthiness, availability or access, price, placement or promotion of food and/or beverages. The modification could occur in any food retail environment, or publicly accessible facility where food was made available. A comparator was required, but there was no limitation on the type. There was no limit on types of outcomes, as it is expected that these are relevant to the type of intervention performed. All original peer-reviewed research, such as cohort, observational, interventional, pre-/post- interventions and randomized controlled trials were considered for inclusion. Study protocols, case reports, opinion pieces, letters to the editor and conference abstracts were not considered to be eligible. Manuscripts had to be in English, available using university-standard library services and be published between 2011 and 2024. These years were chosen as community engagement was more likely to be documented as a process in these latter years.

### Search Strategy

Four electronic databases (MEDLINE (Ovid), PsycINFO (Ovid), CINAHL (EBSCO) and SCOPUS) were searched for related studies. The search strategy was developed by the authors in consultation with a research librarian, based on an adaptation from a previous review that examined food retail environments in rural and remote communities [21]. Database searches were undertaken on 16 February 2023 and rerun on 6 September 2024. A manual search of reference lists from the included studies was undertaken to identify any additional relevant studies. The search terms can be found in Supplementary Materials 1.

### Selection Process

Search results across databases were merged using reference management software EndNote 20 and Covidence systematic review software, and duplicates were removed. At least two authors (YL, LB, TS, LU or SH) independently assessed the title and abstract for all included studies according to the inclusion and exclusion criteria. Screening of full texts for potential inclusion was completed independently by two authors (YL, LB, TS, SH, LU or RM). Conflicts were resolved by discussion, with adjudication by a third author.

### Data Extraction and Synthesis

A data extraction tool was developed in Microsoft Excel. Extraction topics were based on inclusion criteria and data pertaining to the research questions. Demographic and study data extracted included the definition of rural provided, the target population and the food environment objective and outcomes. Data relating to the food environment modification included a description of the food environment, factors contributing to the need for modification, strategies and details of the modification and key findings as presented by individual manuscripts. Community involvement data for extraction included who the contributors were, the method of community engagement, timing of community involvement, roles played and how modifications were influenced by community input. Two authors (YL, LU) extracted data from the full texts identified using the extraction tool, and a second author cross-checked the data (TS, SH, LB, RM). Findings were structured in a narrative synthesis in direct response to the research questions and grouped where similarities between food environments existed. It was expected that included studies had heterogenous study designs, outcomes, and effect measures, therefore a meta-analysis was not intended to be conducted.

### Quality Appraisal of Studies

The quality of included studies was independently assessed by two researchers (LB, TS, SH, RM, LU or LA) using the Mixed Methods Appraisal Tool (MMAT) [22]. This validated tool allows flexibility to assess the quality of a variety of research study types evaluating the methodological quality of five study designs: qualitative research, randomized control trials (RCTs), quantitative non-randomized studies, quantitative descriptive studies, and mixed methods studies. There are two screening questions to determine the feasibility of studies for appraisal by MMAT. Eligible studies were assessed against five questions in relation to study design quality, with “yes”, “no” or “unclear”. Conflicts were resolved by discussion between two researchers. Individual reports from a single overarching study were assessed individually.

## Results

### Study Selection

**Fig. 1:**
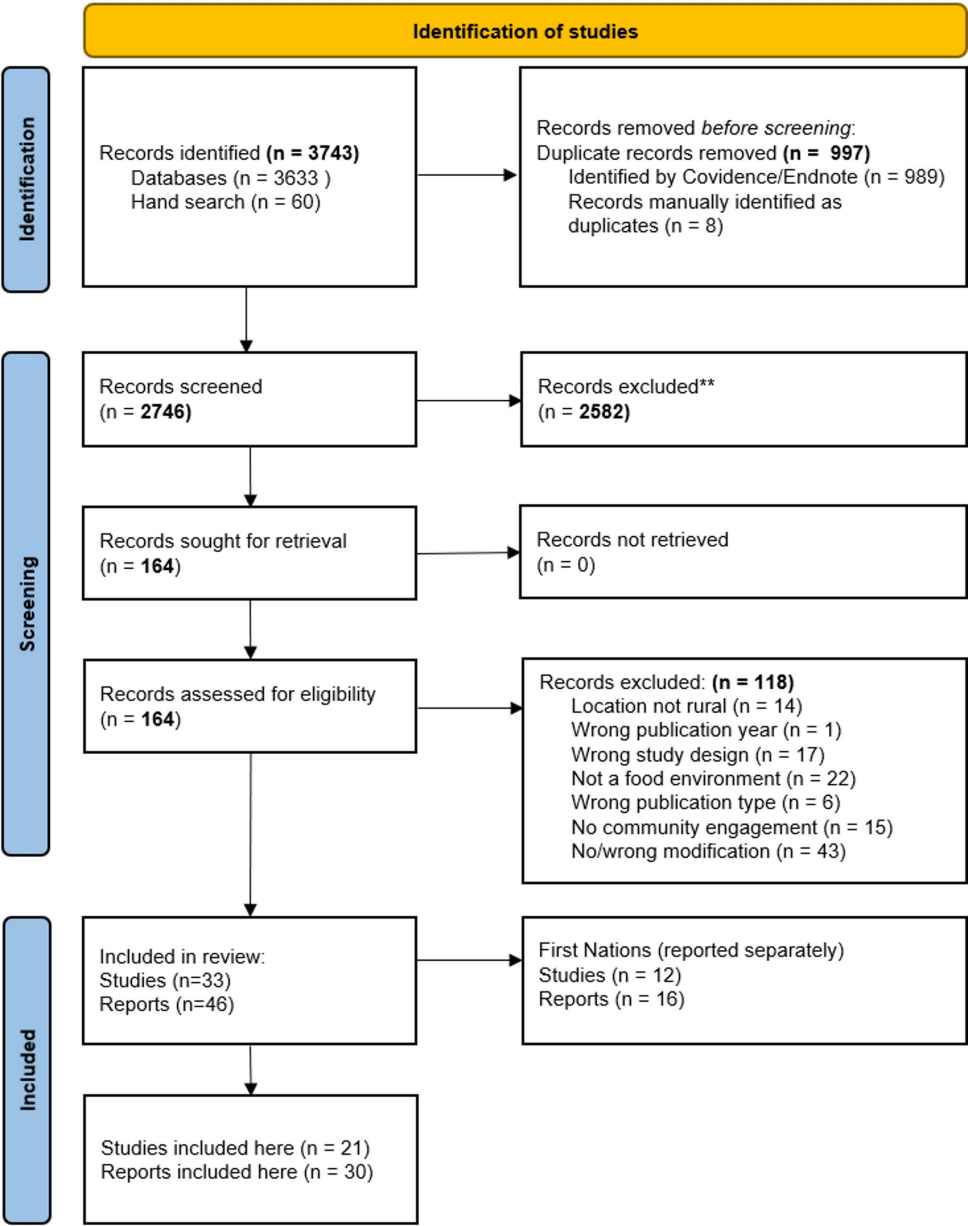
PRISMA flow chart depicting search and selection process

The study selection is shown in Fig. 1. A total of 3633 papers were identified through database searching and 60 through a citation search. 997 duplicates were removed, and 2746 titles and abstracts were screened. A total of 33 studies (45 individual reports) were included after full text review [25–72]. Of theses, 12 studies (16 reports) were conducted with First Nations Peoples [25–42]. Given the complex and sensitive nature of engagement in these communities, the findings of these reports will be reported separately (amendment to the published protocol). Therefore, 21 individual studies (30 reports) were included in this review [43–72]. 

### Study Quality

Out of the 30 included reports, five reports [43, 47, 49, 56, 61] did not meet the MMAT screening criteria and three reports [41, 63, 64] had study designs unsuitable for the MMAT, therefore could not be assessed for quality with this tool. No particular study design fared better than another in the MMAT quality rating, due to a lack of clarity in the methods of some studies, which may be related to the complexity of the intervention and study design, populations affected, or food environment being described. See Table 1 for quality assessments of individual reports.

**Table 1:**
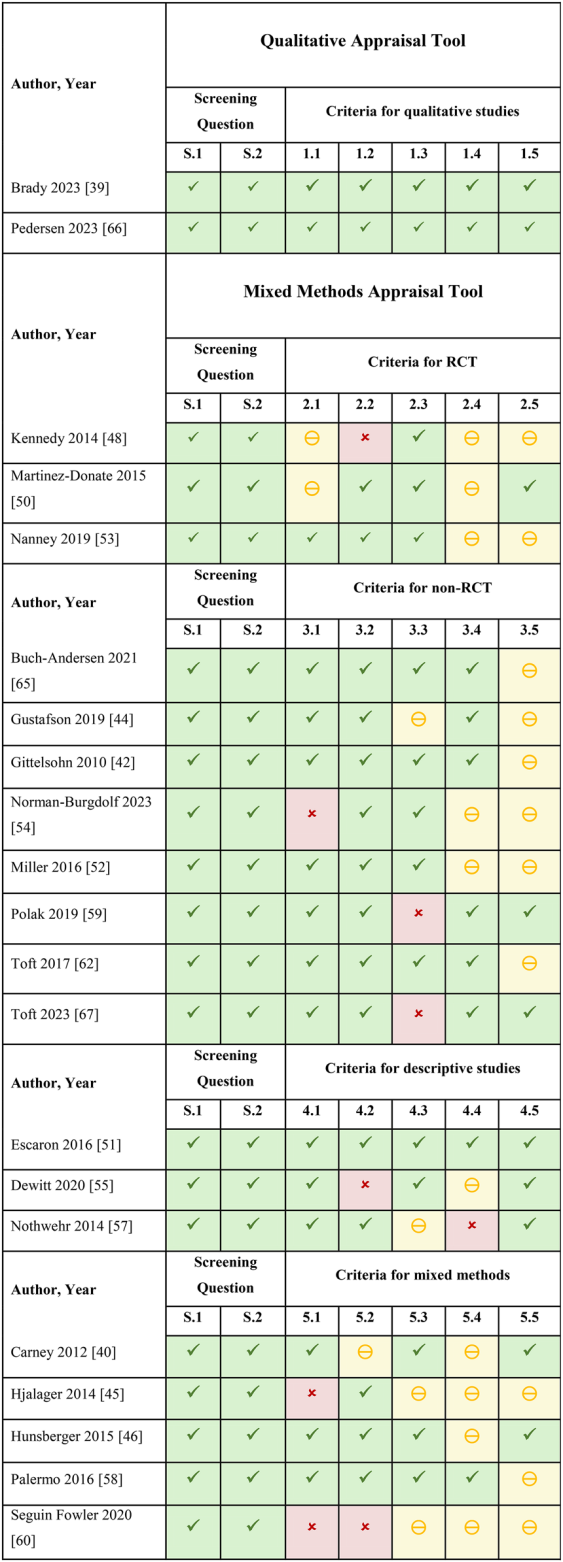

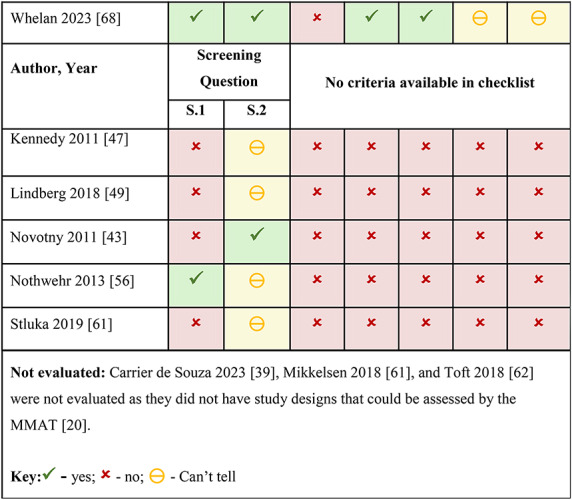
Quality appraisal of included studies/programs using the mixed methods appraisal tool (MMAT) [22]

### Study Characteristics

Studies identified were predominantly completed in the USA (*n* = 15) [39, 40, 46–48, 50–58, 60, 61], with two from Australia [58, 68] and Denmark [45, 66–71], and one from Israel [59] and Italy [41] respectively. As expected, study designs were heterogeneous, ranging from RCT (*n* = 3) [48, 50, 53] to descriptive studies (*n* = 1) [61] (see Table 2). Primary outcomes were also heterogeneous, with *n* = 7 relating to fruit and/or vegetables [40, 44, 47, 48, 54, 58, 61, 62, 67] and *n* = 8 reports evaluating interventions [49, 50, 56, 57, 60, 62, 65, 67].

### Community Involvement in Food Environment Modifications

The local community contributors varied across different settings (see Table 3). Community members from the local area (*n* = 10) [39, 40, 44, 47, 48, 52, 54, 59, 66–72], and store and restaurant owners (*n* = 5 [39, 42, 43, 58, 66–71] and *n* = 5 [53–57] respectively) were the most frequently mentioned local co-design contributors. Most studies (*n* = 16) [39, 41, 48–53, 62–72] mentioned that there were local contributors involved before interventions were implemented, specifically during the planning of intervention. Four studies [40, 41, 54, 66–71] stated that local contributors were involved throughout the intervention, however, a further four studies [42, 43, 49, 52] did not include details of when local contributors were involved in their interventions.

**Table 2:** Demographics, objectives, and outcomes of included studies

Author & Year	Program/ Study title	Country	Food-environment related objective	Primary Outcome(s)	Study design	Comparator	Target population & their location	Rural definition
Brady 2023 [39]	University of Minnesota Supplemental Nutrition Assistance Program-Education (SNAP-Ed), *Nudging to Health* and *SuperShelf*	United States	To identify promising practices from rural food pantries that effectively and respectfully served their shoppers and determine current and future needs.	How emergency food service providers serving rural areas work to meet the needs of their communities.	Qualitative	No comparator required	Food pantry staff, volunteers and shoppers in Greater Minnesota	Rural-Urban Continuum Codes and Rural-Urban Community Codes.
Carney 2012 [40]	Community gardening project for Hispanic community	United States	Study the impact of a community gardening program on vegetable intake in rural Oregon community	Post-intervention vegetable intake and food security concerns	Pre/post study using mixed methods	Baseline data collected pre-intervention	Latino population living within the rural Oregon community	Author-designated as rural
Carrieri de Souza 2023 [41]	Food networks and agroecology in the Province of Trento *Nutrire Trento Project*	Italy	To identify and understand Civic Food Networks (CFNs) where farmers play an active and organized role alongside other actors within the agrifood system.	Evaluation of the promotion of agroecology descriptors (including biodiversity, resources efficiency and production for self-produced food)	Case study	No comparator required	Farmers and consumer families from the Province of Trento	Author-designated as rural
Gittelsohn 2010 [42] & Novotny 2011 [43]	Healthy Foods Hawaii (HFH) project	United States	Describe the development and implementation of HFH project aimed to improve children’s dietary behavior to prevent child obesity	Children’s food intake and evaluation of intervention	Pre/post intervention study	One control/comparison community on each island - the control group share similar ethnic and income distribution with the intervention group	Children and adult caregiver in rural Hawaii	Author-designated as rural
Gustafson 2019 [44]	Plate it Up Kentucky	United States	Determine effectiveness of the program by assessing pre/post dietary intake	Changes in fruits and vegetables intake	2 cross-sectional studies	Baseline data collected pre-intervention	Adults residents in 6 counties (Clinton, Elliott, Letcher, Lewis, Logan & Martin)	US Department of Agriculture Rural Codes 7 or higher
Hjalger 2014 [45]	MarBioShell	Denmark	Examine how, and with what results, guests at a science center can contribute to foods based on blue mussels	Evaluation of user-driven methodology to introduce mussels to museum guests	Mixed methods	Qualitative data & sales figure	Visitors at Fjord & Baelt and guests at cafeteria from Kerteminde and the larger Funen region	Author-designated as rural
Hunsberger 2015 [46]	Calorie Labelling in rural middle school	United States	Investigate impact of calorie posting at the point-of-purchase in a middle school cafeteria	Changes in gross calorie consumption and fat intake	Mixed Methods	Baseline data collected pre-intervention	Grades 6–8 students in Jefferson County Middle School (JCMS)	Author-designated as rural
Kennedy 2011 [47] & 2014 [48]	People United to Sustain Health (PUSH)	United States	Evaluate the effectiveness of PUSH (a CBPR intervention) in promoting healthy lifestyle among community in Lower Mississippi Delta (LMD)	Changes in anthropometric measurements, blood profile, fruit and vegetable consumption	Randomized trial with pre/post measures	49 participants within the same community randomized into treatment group (*n* = 26) and control group (*n* = 23). Treatment group received 24 weekly visits to the “Rolling Store” plus nutrition education classes. The control group received family coping classes.	Adult men and women (18 years or older), residents of Franklin Parish (Louisiana) with a BMI of 23–45, one person per household	Author-designated as rural
Lindberg 2018 [49]	Heart of New Ulm (HONU) restaurant programme	United States	Describe the implementation and changes made in rural restaurant food environment through community-based interventions	Adoption and implementation of healthy practices	Pre/post-evaluative design	Each participating restaurant has a personalized baseline assessment report on status of each healthy practice domains	Residents residing in the 56,073 zip code at New Ulm, south-west Minneapolis/St Paul metropolitan area	Author-designated as rural - New Ulm located about 160 km south-west of Minneapolis metropolitan areas
Martinez-Donate 2015 [50] & Escaron 2016 [51]	The Waupaca Eating Smart	United States	Pilot-test a community-level intervention to improve the nutrition environment of rural food stores and restaurants	Evaluation of RE-AIM to inform future trial	Pilot RCT	One county with similar characteristics as the intervention group	Rural adult residents in 2 Midwestern U.S counties	Author-designated as rural
Miller 2016 [52]	Community adaptations to food desert in rural Appalachia	United States	Investigate how a small rural community adapted to an impending food desert	Comparison of food shopping behaviors, restaurant activities, community market response and Green Grocer sales activities before and after emergence of food desert	Pre/post evaluative design through survey activities	Community members act as their own control	Community in Alderson, West Virginia	Author-designated as rural
Nanney 2019 [53]	School Breakfast Program (SBP) in 16 rural high schools in Minnesota	United States	Evaluate a school-based intervention designed to increase SBP participation	Change in participation between baseline and follow-up measures (1 year)	RCT	4 schools act as control (later received delayed intervention 1 year after result collection)	Students in Year 9 & 10	Author-designated as rural
Norman-Burgdolf 2023 [54] & DeWitt 2020 [55]	Community driven interventions in a rural Appalachian county	United States	Determine the impact of community-driven evidence-based initiatives and policy, system, and environmental (PSE) changes facilitated by Extension over 3-year period on fruit, vegetable and beverage intake	Fruit and vegetable intake	Prospective cohort study	Baseline data at the beginning of study in Fall 2019	Adult residents in Martin County, Kentucky	Author-designated as rural
Nothwehr 2013 [56]	Promoting healthy choices in non-chain restaurants	United States	Examine the reach, effectiveness of the signs promoting healthy choices at restaurants on ordering behavior	Evaluation of reach and effectiveness	Mixed Methods	Baseline data collected pre-intervention	Customers in 4 owner-operated restaurants in rural Iowa	Author-designated as rural
Nothwehr 2014 [57]	State-wide dissemination of a rural, non-chain restaurant intervention	United States	Examine the adoption rate of the program and to describe implementation and maintenance issues.	Evaluation of adoption, implementation and maintenance at 3-month, 6-month, 12-month, 18-month follow-up	Mixed Methods	Brief telephone interview prior to implementation	100 restaurant owners in the Mid-West state	Based on Hall et al. 2006 as having less than 50,000 residents
Palermo 2016 [58]	Initiative in small rural stores to improve access to fruit and vegetables	Australia	Evaluate a health promotion fruit and vegetable access initiative in small communities across rural Victoria	Impact of initiative and determine changes in access to fruit and vegetables in participating stores	Mixed methods pre-post evaluative design	Baseline data of variety and price of fruit and vegetables	21 small towns across two rural Victorian Local Government Areas, total population 12,139	Australian Standard Geographical Classification - Remoteness Area (ASGC-RA)
Polak 2019 [59]	Community culinary coaching program in a rural kibbutz cafeteria	Israel	Describe the programme’s impact on the alignment of cafeteria food with Mediterranean-style diet and diners’ consumption habits and satisfaction	Mediterranean index, diner’s consumption habits and satisfaction	Non randomized, controlled design with pre/post evaluation	Two Kibbutzim with a similar socio-demographic and health parameters as the intervention group	Adults and children in rural kibbutz (Intervention: 493 adults, 214 children, Combined control sites: 487 adults and 206 children)	Author-designated as rural
Seguin-Fowler 2020 [60]	The eHeart Study	United States	Evaluate the effectiveness of the adaptation of the HEART Club (Change Club) intervention into a web-based format specifically assessing the implementation and feasibility of the eHeart approach	Positive and challenging aspects of the eHeart project, recommendations for improvement	Descriptive with qualitative	No comparator required	3 rural towns in 3 US states. Two focused on students in middle school, one focused on students in transition (homeless, youth, couch surfing, living in cars).	Author-designated as rural
Stluka 2019 [61]	Gardening for Health	United States	Describe how community garden were used to provide access to healthy food, increase access to nutrition	Characteristics of garden (garden size, items planted, visitors)	Descriptive	Each garden acts as its own control	Local communities in South Dakota	Author-designated as rural
Toft 2018 [63], Mikkelsen 2018 [64], Buch-Andersen 2021 [65], Toft 2023 [67], Toft 2017 [62] & Pedersen 2023 [66]	Project SoL	Denmark	To evaluate change from multicomponent intervention using various domains including health behaviors, purchasing patterns, overweight and obesity among children.	Multi-component including: Behavior change – diet, shopping and eating practices BMI and waist circumference of children Supermarket sales volumes of fruit, vegetables, fish and whole grains.	Mixed-methods, quasi-experimental study	Intervention municipality of Bornholm matched with control municipality of Odsherred. Three-year follow-up after intervention ceased.	Families with children enrolled in the participating childcare centers (3–6 years) and primary schools (6–8 years)	Author designated as – regional, small towns
Whelan 2023 [68]	Health Promoting Café in rural Victoria	Australia	Evaluate proportion of sales attributable to each of the traffic light system, perspectives of customers and staff use and sustainability of the system	Monthly sales data in the cafe coded to the traffic light system	Mixed methods	Sales data	Rural Northwest Health Service staff (200), aged care facility residents (60) and an average of 35 allied health clients attending the service per day	Author-designated as rural (360 km from the nearest state capital of Melbourne, Victoria, 6658 people spread over 7326 square kilometers)

Abbreviations:

CBPR – Community Based Participatory Research

RCT – Randomized Controlled Trial

US – United States

**Table 3:** Community involvement described in the included studies

Author & Year	Local contributors	Other collaborators	What approach was used for engagement?	When did the local contributors get involved?	What roles did the local contributors play?	How did community involvement influence modifications?
Brady 2023 [39]	• Community members • Food pantries (volunteers and staff) • Food pantry directors	SNAP-Ed educators	Place-based approaches involving community members to develop evidence-based programs adapted to unique community priorities	Food pantries that had worked with SNAP-Ed for > 10 years were involved in the study.	• Food pantry directors assisted with organizing focus groups	Not described
Carney 2012 [40]	• OHSU group • Community groups • Participating families	Project health promoter	Community based participatory principles	Local contributors were involved throughout the intervention	• Community groups: organize all community meetings and interactions with participating families; translate and adapt materials for appropriate grade level, plain language and health literacy • OSHU group: develop educational materials about the harms of pesticides and how to avoid them	Not described
Carrieri de Souza 2023 [41]	• Farmers, trade unions, consumers, institutions, associations, schools, universities and research institutes.	Trento’s municipal government, Department of Sociology and Social Research of the University of Trento.	Multi-stakeholder roundtable discussions.	Local contributors were involved over the course of the project (4 years).	• Regular meetings to develop an interactive map of short food supply chain initiatives. • Development of other initiatives including a pilot project during the COVID-19 pandemic that aimed to bring together consumer families and farmers for direct sale and home delivery.	Not described
Gittelsohn 2010 [42] & Novotny 2011 [43]	• Store owners and managers • Food producers and distributors	HFH project staff	Community workshops were held to develop HFH intervention messages specifically on local foods and agricultural products consumed by children (no further details provided on who were involved in the workshop)	Not described	• Food producers and distributors: increase availability of HFH targeted products, provide product and/or promotional materials for taste tests and cooking demo • Store owners and managers: Hold cooking demonstrations and taste test in their store, display promotional materials. No further details provided	Community was consulted for the development of HFH intervention messages to find out local foods and agricultural products consumed by children
Gustafson 2019 [44]	• Residents in the county	Students & staff from University of Kentucky and FCS Extension agents	Residents’ engagement was facilitated by FCS agents, no further details described	Residents were engaged during the planning of intervention	• Residents in the county advised on community needs	Community needs were assessed and insights given by locals helped to prioritize targeting of grocery stores and farmers markets.
Hjalger 2014 [45]	• Visitors • Cafeteria staff • Cafeteria manager	External social researchers	Not described	• Staff and manager were involved during the planning of intervention • Museum visitors were invited to participate in the experimental set-up	• Cafeteria staff: prepare mussel soup, inform customers on ingredients & obtain feedback from guest • Cafeteria manager: overlook the operation of cafeteria • Visitors: participate in activities at the science museum	Not described
Hunsberger 2015 [46]	• Members from the Research Partnership	Research staff	Not described	Members from the Research Partnership: during the planning of intervention	No further details provided	Not described
Kennedy 2011 [47] & 2014 [48]	• Franklin Parish community residents and key community stakeholders	Owner of the Rolling Store and research investigators	CBPR principles	Franklin Parish community residents and key community stakeholders: from inception and during the planning of intervention	• Franklin Parish community residents and key community stakeholders: assess the health problems in their community, adapt previous research done by the research team to plan the current intervention, hiring and training, recruitment and retention, assist with data collection and submission	CBPR allow community residents to work collaboratively with research partners to identify and prioritize health issues and develop intervention strategies that were considered to be beneficial for the communities.
Lindberg 2018 [49]	• Restaurant owners • Local dietitian	Not described	Local dietitian called all restaurants and made a time to meet one-on-one with interested restaurant decision makers	Not described	• Restaurant owners: implement program intervention in their restaurant • Local dietitian: provide consultations to restaurants, no details on what the consultation was about	Not described
Martinez-Donate 2015 [50] & Escaron 2016 [51]	• Operators • Local registered dietitian (RD) • One well-known member in the community	Program staff and coalition staff	Coalition staff selected outlets to approach based on likelihood to participate in healthy eating campaign, then recruit outlets using semi-structured interviews	• Operators: during the intervention • Local RD: during the planning of intervention • Well-known community member: during the intervention	• Operators: Choose possible interventions from the menu to implement in their outlets • Local registered dietitian (RD): analyze menu items at restaurants, then create “WES approved meals” • Well-known member of community: WES spokesperson	Coalition staff used the results of the initial formative assessment to inform intervention planning, outlined a process that included feedback from community stakeholders to promote healthy eating within the community.
Miller 2016 [52]	Community members	Research team	CBPR principles	Not described	• Community members participated in the research effort	Community based participatory research with members of the Alderson Community Food Hub Board of Directors. No further details provided
Nanney 2019 [53]	• SBET - consisting of an existing school wellness committee and/or a new committee and included multiple stakeholders • Students • School principals, food service director	Not described	Information webinars for interested school personnel, mainly principals and food service directors.	• SBET: during the planning and execution of intervention • Students: during the planning and execution of intervention • School principals, food service director: before intervention	• SBET: meet up before implementation of changes to the SBP and after implementation had begun to discuss assessment, goal setting and developing plan/monitoring strategies **•** Students: work with a marketing firm to develop and implement a school-specific marketing campaign. Identify existing perceived barriers to SBP participation and develop marketing theme unique to school setting and student preferences • School principals, food service director: sign MOU before implementation, committing to three overarching intervention components: a grab-and-go breakfast outside of the cafeteria, allowing eating in the hallway and develop a student-led marketing campaign.	Student-developed marketing campaign were able to identify perceived barriers of the program, and develop a marketing theme suited to the school setting and student preferences
Norman-Burgdolf 2023 [54] & DeWitt 2020 [55]	Cooperative Extension Service (Extension), local Wellness Coalition & “Community based organizations”	Center for Disease Control and Prevention’s High Obesity Program & land grant Universities	**•** Annual focus groups with local residents and stakeholders **•** Wellness Coalition had quarterly meetings and regular correspondence via email and newsletter.	Wellness Coalition involved prior to establishing local partnerships	**•** Established partnerships **•** Provided input and direction for all aspects of the project	Unclear
Nothwehr 2013 [56]	Restaurant owners	Research staff	Research staff identified restaurants at nearby towns. No further details provided.	Restaurant owners: after the list of modification strategies were designed by research staff	• Restaurant owners: decide or edit the list of healthy options based on the suggested list to be put up on the plastic signs	In a pilot test of the intervention in a similar restaurant, a list of healthy options was developed for the signs based on what the owner stated would be acceptable.
Nothwehr 2014 [57]	Restaurant owners	Research staff	Research staff contacted restaurants by phone	Restaurant owners: after the list of modification strategies were designed by research staffs	• Restaurant owners: decide or edit the list of healthy options based on the suggested list to be put up on the plastic signs	In a pilot test of the intervention in a similar restaurant, a list of healthy options was developed for the signs based on what the owner stated would be acceptable.
Palermo 2016 [58]	• Local participating retailers • Project officers employed by the local government	Trained student dietitian and research staff	Set up a project team consisting of a local project coordinator, council member, health sector representatives and department of health representative. A program logic model was used to facilitate agreement on the focus of the evaluation with multiple stakeholders, to inform the evaluation approach and identify data that needed to be collected.	• Project officers: during the planning of intervention • Local participating retailers: during the intervention	• Project officers: identified appropriate set of options for fruit and vegetable display and incentives for retailers, developed package of support for retailers to assist with implementation. Involvement of local participating retailers in determining incentives to be used in their store. • Local participating retailers: take on incentives that were most suited for their store’s needs to increase sales of fruits and vegetables.	Staff at Local Shire Council to design and implement a local social marketing campaign targeting consumption of fruit and vegetables, local shire councils to facilitate training and ongoing support for the program.
Polak 2019 [59]	• Intervention community administration • Community steering committee • Locals (pre-school staff, kitchen staff, community members)	Research team	CBPR principles	• Intervention community administration: during the planning of intervention • Community steering committee: during the planning of intervention • Locals: during intervention when culinary coaching was provided	• Intervention community administration: identify priorities and key principles for intervention • Community steering committee: review goals set for implementation of the intervention and review community feedback • Locals: attend training programs on freshly prepared Mediterranean-style diet and how to impact nutritional change among children	Community representatives act as equal partners in determining the desired process, nutritional goals and pace of adopting them during intervention.
Seguin-Fowler 2020 [60]	• Local extension educator (CC facilitators) • Community residents • Local web developer	• Research team • Extension educator	Extension educators were invited to disseminate the eHeart website and application form to local community organizations and groups	Community residents: during the development of intervention strategies with the guidance of extension educators	• Local extension educator (CC facilitators): facilitate the implementation of program • Community residents: guided the intervention strategies of the program • Local web developer: develop website according to the needs of the community	Community assess their surroundings, identifying issues, developing and enacting a plan for built environment change
Stluka 2019 [61]	• Local wellness coalition • Seasonal garden coordinators	• SDSU Extension staff members	Not described	• Local wellness coalition: established during the planning of intervention • Seasonal garden coordinators: during the intervention	• Local wellness coalition: recruit and engage community members and raise awareness about coalition efforts • Seasonal garden coordinators: assist community members with gardening, recruit community members to participate in activities, train volunteer groups, maintain garden records	Seasonal garden coordinators assist the community with their gardening efforts and encouraged more community members to participate in this project.
Toft 2018 [63], Mikkelsen 2018 [64], Buch-Andersen 2021 [65], Toft 2023 [67], Toft 2017 [62] & Pedersen 2023 [66]	•Local stakeholders including action groups, local government, primary schools, after-school centers, childcare centers, supermarkets, media, NGOs, stakeholder forum, and experts in nutrition, cooking, recreation and physical activity. •Target groups children and families	Steering committee, Independent advisory board, research group, executive committee, employment of local coordinator.	Action research approach and a participatory intervention strategy to mobilize local communities for public health action. Local capacity building.	Initially inviting and engaging local partners prior to intervention to inform development of intervention components.	• Inform development of interventions •Stakeholder analysis •Interventions implemented collaboratively with stakeholders	The intervention development was based on a balance between what was brought forward by the participatory groups, the evidence base and what was already up and running and locally prioritized. Activities varied by setting depending on the ideas of stakeholders in different communities.
Whelan 2023 [68]	• Community leaders • Café staff	• Research team	Community food environment audit with the involvement of community leaders	• Community leaders: during the planning of intervention • Café staff: No details included.	• Community leaders: involved in the creation and continuation of health-promoting café to advocate for healthier food provision	Not described

Abbreviations:

CBPR - Community Based Participatory Research

CC – Change Club

FCS - Family Consumer Science

HFH - Healthy Foods Hawaii

OHSU - Oregon Health & Science University

MOU – Memorandum of Understanding

NGO – Non-government organization

SBET - School Breakfast Expansion Team

SDSU - South Dakota State University

WES - Waupaca Eating Smart

### Influence of Community Involvement on the Modifications Made to the Rural Food Environment

Twenty-one reports [42, 44, 47, 48, 54–58, 60–71] mentioned the involvement of community and how this involvement influenced the food environment modification to varying degrees. Five studies included local contributors as consultants for the programs to identify and advise on community needs [44, 47, 48, 54, 60, 66–71]. For example, the Danish study Project SoL, involved the community in the project’s organizational structure, alongside key research institutions and government organizations focused on local ownership and sustainable integration of interventions [66–71]. Eight studies [40, 42, 43, 50, 51, 53, 65–71] mentioned that some local contributors were acting as advocates for the programs where their role was to raise awareness about the program or in developing promotional or educational materials specific to their community. In a novel example, the Waupaca Eating Smart (WES) intervention had a local “celebrity” eat at WES-aligned restaurants during the intervention period and wrote about his experiences in the local media [50]. Local contributors in three studies [60–62] were only responsible for adopting the strategies after they had been designed, indicating only later levels of community involvement in the interventions. One study [46] did not include any clear details on the role of local contributors in their program.

### Food Environment Targeted for Modification

Food environments targeted for interventions included restaurants (*n* = 3) [49, 56, 57], schools (*n* = 3) [46, 53, 60], community garden/home garden (*n* = 3) [40, 61], food stores (*n* = 2) [42, 43, 58], cafeteria (*n* = 1) [45], café (*n* = 1) [68], a Rolling Store (*n* = 1) [47, 48], food pantries (*n* = 1) [39], food supply chains (*n* = 1) [41] and a kibbutz (*n* = 1) [59]. Some interventions targeted more than one food environment, such as restaurants and food stores [44, 54–56, 66–71].

### Modifications Made to Rural Food Environments

The types of food environment modifications varied across interventions. Eight studies reporting positive outcomes involved the use of promotional strategies in their programs along with other modification strategies [42, 44, 45, 51–53, 58, 66–71]. Another common food environment modification strategy was to increase availability of particular types of food (*n* = 7) [43–46, 49, 53, 58, 59, 65–71]. Four studies used a single modification strategy such as point-of-purchase interventions [46, 68] or promotion [56, 57]. The most common combination of food environment modification was to increase the availability and access to foods [43–45, 52, 54, 59, 61]. Three studies [44, 50, 51] used point-of-purchase interventions and promotion as their modification strategies, while some adopted an integrated approach that used multiple food environment modification strategies. For example, the People United to Sustain Health (PUSH) study [47, 48] focused on increasing access and availability of healthier items, providing subsidies and giving out promotional materials related to the program. Twelve studies [39, 40, 42, 48–53, 58, 59, 65–71] reported positive study outcomes as a result of the food environment modifications that were implemented (see Table 4).

**Table 4:** Details of chosen food environment, food environment modifications made and key findings from included studies/programs

Author & Year	Factors leading to the need for intervention	What is impacting the rural food context?	Description of food environment	Food environment modification strategies	Details of modifications	Key findings from modifications
Brady 2023 [39]	Emergency food system’s effectiveness in addressing shoppers’ food and nutrition security is limited.	Barriers to nutritious food access due to reliance on donations, structural barriers (e.g. transport) and stigma.	7 food pantries serving residents of Greater Minnesota	• Increase availability • Increase access • Point-of-purchase	*Increase access and availability of healthy foods* -Increase the capacity of food pantries to supply healthy foods by providing support and education to staff. - delivery/mobile food pantries to improve geographic accessibility. *Point-of-purchase* -Increase the capacity of food pantries to increase the prominence and appeal of healthy foods through welcoming spaces, client choice and behavioral economics.	Food pantries met their client’s needs and provided healthier options despite barriers including transportation, organizational policies, stigma, food availability, financial, physical, labor and technical capacity.
Carney 2012 [40]	Risk of food insecurity is higher among Hispanics which commonly leads to associated health outcomes.	Not described	Home garden, no further details provided	• Increase availability	Hispanic families enrolled in the project will establish home gardens and will be provided with resources, materials, volunteer support. Education sessions to address any concerns faced.	Frequency of adult and children’s vegetable intake increased (*p* < 0.05), while concerns regarding food security decreased (*p* < 0.05)
Carrieri de Souza 2023 [41]	Current food systems are unsustainable in socio-environmental terms.	A consumer movement around food is present in the area being studied (Gruppi di Acquisto Solidale or Solidarity Purchase Groups). Grassroots initiative for consumers to buy directly from local producers.	Civic Food Networks (CFNs) and short food supply chains of agroecological food	•Increase availability •Increase access	Trento’s municipal government started a process to support policies and actions to increase sustainable food production. Food deliveries from farmers to consumer families. Farmers markets supported.	Farmers involved in CFNs produce 60% of food in their diet, compared to other farmers (33%). Increased networking capital between farmers and consumers.
Gittelsohn 2010 [42] & Novotny 2011 [43]	High childhood obesity prevalence, low income levels with > 75% of population below poverty level.	Not described	5 intervention stores which are primary sources for food purchasing in the community	• Recipe modifications • Promotion • Increase availability	*Recipe modifications* -Local recipes were modified to improve healthiness *Promotion* -In-store posters, educational displays and shelf labels were posted in various food, health and community locations. *Increase availability* -Local producers and distributors provided promoted products which were specific to each phase of the intervention	Intervention was effective in improving healthy food knowledge and increased the consumption of healthy foods by children. A high to moderate dose and reach of the intervention was achieved overall, but fidelity of intervention was moderate.
Gustafson 2019 [44]	A need to understand key drivers of obesity and to identify opportunities for obesity prevention in rural communities with > 40% obesity prevalence, poverty rates 25.7–35.7%, food insecurity rates 15.2–20.1% & unemployment rates 9.6–17.3%.	• High produce costs resulting from geographic remoteness.	Grocery stores and farmers markets, targeting at least one supermarket or supercenter in each county.	• Promotion • Recipe modifications • Point-of-purchase • Incentives	*Recipe modifications* -Healthy recipes with samples at check-out caps *Promotion* - Intervention promotion on grocery carts with recipes & logos displayed *Incentives* -Tote bags and gel packs for trying samples - $5 gas cards to encourage shopping at farmers markets	Between years 1–2, increases were seen in: - fruit servings/day: 2.71 to 2.94 - vegetable servings/day:2.54 to 2.72 - shopping at farmers market once a week: 7–12%.
Hjalger 2014 [45]	Locals were not aware of the importance of mussels, therefore drives the need for education	Kerteminde Bay is unaware of its capacity to produce good quality mussels	Cafeteria associated with Fjord & Baelt (the science museum)	• Promotion • Subsidies	*Promotion* - Introduction of new food item (mussel soup) to the cafeteria - Display of poster about the soup *Subsidies* - Price subsidized on mussels soup to encourage cafeteria manager to market the soup	Process has successfully engaged people. 44 soup portions were sold over 2 weekends. Taste trials identified interest in mussels
Hunsberger 2015 [46]	High obesity prevalence amongst 6th to 8th grade students at JCMS	Not described	School situates in a low income community and participates in the USDA National School Lunch Program	• Point-of-purchase	Posting calorie labels on cafeteria menu	Calorie consumption and fat intake among students decreased post-intervention (*p* < 0.05). Results correspond with qualitative responses from students.
Kennedy 2011 [47] & 2014 [48]	High prevalence of adult obesity and poverty rate	Not described	African American Baptist church located in Winnsboro, Louisiana- The ‘Rolling Store’	• Increase availability • Increase access • Subsidies • Promotion	*Increase access and availability of healthy foods* - The ‘Rolling Store’ will come on the same day each week to sell quality fresh fruits and vegetables to participant at the church *Subsidies* - Budget of $213 per week was allotted for purchasing fruits and vegetables to stock up the store *Promotion* - Weekly handouts with a featured fruit and vegetable given to participants, recipes included	No significant difference in mean anthropometric measurements over 6 months between both groups, weight was maintained. Mean fruit consumption increased over 6 months for treatment group (*p* < 0.05) but mean vegetable consumption remained the same between the groups.
Lindberg 2018 [49]	Obesity, metabolic syndrome, and low fruit and vegetable intake are key health risks within the population	Not described	33 community restaurants including sit-down restaurants with table service, fast-food restaurants, and carry-out or delivery setting	• Increase availability • Promotion	*Increase availability* - Offering of non-fried vegetable side dish, half-portion entree, low fat/ sugar-free beverages, healthier preparation methods for sandwiches/burgers *Promotion* - Promote healthier options on menu/table tent for customers to see prior to ordering - Social marketing campaign to educate consumers and increase demand for healthier menu selections	Adoption and implementation improved from 22–38% in restaurants, especially those that were independently owned. The healthy practice showing most improvement were: availability of non-fried vegetables, fruits, smaller portions and whole grains.
Martinez-Donate 2015 [50] & Escaron 2016 [51]	Absence of other major healthy eating initiatives among the targeted population. No further details provided.	Not described	7 restaurants and 2 food stores	• Point-of-purchase • Promotion	*Point-of-purchase* - Labelling healthier items in restaurants and food stores *Promotion* - Promote healthy family meals on recipe cards - Display promotional items around premise - Display WES logo near restaurant entrance - Promote healthy bundled meal ideas	Findings demonstrate interventions are feasible and acceptable but low levels of effectiveness.
Miller 2016 [52]	Rural Appalachia has some of the highest rates of CVD and mortality in the USA. The poverty rate in Alderson is much higher than the national poverty rate in the country	Closure of the only grocery store in the county led to poor accessibility of fresh food produce for the community	The closest grocery store for Alderson residents is situated 17.7 km away, there are only 4 fast food restaurants and 2 convenience stores in the area after closure of a full-service grocery store.	• Increase access • Increase availability	*Increase access and availability of foods* - Setting up of the Green Grocer - Community gardening	Home gardening increased by 21% after closure of full-service grocery store. However, no significant changes before and after the set-up of Green Grocer, 77% of the population did most shopping at a store at least 17.7 km away from home due to expensive food items at the Green Grocer.
Nanney 2019 [53]	Student participation in SBP is low in high schools, which breakfast skipping is associated health concerns	30% of US adolescents not consuming breakfast, with low SBP participation rate (< 20%)	Rural schools located outside of the seven-county Twin Cities metropolitan area, not having a grab-and-go reimbursable school breakfast option, low SBP participation (< 20%), > 500 student enrolment, > 10% student minority enrolment	• Increase availability • Promotion	*Increase availability of breakfast* - Provide grab & go breakfast outside of cafeteria, (second chance breakfast), allow students to eat breakfast in hallways *Promotion* - Create promotional strategies (student-developed videos, school newsletter, student orientation, taste testing).	Statistically significant result (*p* < 0.03) shown between intervention and control groups suggested increased level of SBP participation rate among students − 3% increase in intervention group and 0.5% increase in control group.
Norman-Burgdolf 2023 [54] & DeWitt [55]	High obesity prevalence and lower life expectancy compared to state and national average, persistent poverty, food insecurity, high disability rates	Poor access to healthy food options and resource poor neighborhoods in rural Appalachian	3 Martin County food pantries, 3 local faith-based organizations, Martin County Farmers’ Market, 5 local gas stations, 2 local schools and senior citizens center	• Increase availability • Increase access	*Increase access to and availability of fruits and vegetables* - Increase capacity of local food pantries by making improvements to state and local program/systems, work with food vendors, distributors, and producers to enhance healthier food procurement and sales	Increase in servings of vegetables with a decrease in fruit and legume consumption. Authors highlighted that there may not be a direct causal association between programs and dietary choices due to external reasons [52].
Nothwehr 2013 [56]	Not described	Not described	4 owner-operated, full-menu, sit down Mid-western restaurants in separate small towns in rural Iowa. All had been in business for > 1 year and had no customer base overlapped with the others	• Promotion	- Placement of plastic signs at each table listing options for making food orders more healthful - Placement of sign in entryway or front window at restaurant to promote the initiative	50–60% of customers noticed the nutrition information, however 34% report the signs influenced their order. Intervention resulted in small behavior change.
Nothwehr 2014 [57]	Not described	Not described	100 restaurants chosen were those of family style, sit-down restaurants, with a full menu, not primarily specialty/ethnic, in rural counties, opened for at least 1 year	• Promotion	- Placement of plastic signs at each table listing options for making food orders more healthful - Placement of sign in entryway or front window at restaurant to promote the initiative	28% of the restaurants adopted the program. 14 out of 28 restaurants maintained using table signs at 18-month follow-up point.
Palermo 2016 [58]	Rural population often has poor geographical access to healthy food (e.g. access to fruits and vegetables)	Poor geographical access and range of fruit and vegetables available within the population	Stores in towns with a total population of less than 900 people	• Increase availability • Increase access • Promotion	*Increase access and availability* - Project officer offered incentives aimed to increase sales of fruits and vegetables in stores (refrigerator/refrigerator repairs, ambient displays, signage, cooking demonstrations, shopper giveaways, fruit and veg boxes, recipe cards, loyalty cards, business innovation expertise), retailers then choose the incentives most suited to their store needs *Promotion* - Local social marketing messages were created	Initiative was effective in improving the availability of fruit and vegetables in small stores. Access may have improved for some subgroups (greatest change observed in stores that did not previously sell fruits and vegetables) but sustainability is unlikely without continued resourcing.
Polak 2019 [59]	Improving eating behavior is identified as a major Israeli public health priority	Not described	Kibbutz is a communal settlement where people live as a cooperative community, sharing income and expenses and eating food that is prepared in the community cafeteria	• Increase availability • Increase access	*Increase access to and availability of healthy foods* - Conduct community culinary coaching program focusing on freshly prepared Mediterranean-style diet to provide healthier options in the communal cafeteria and pre-school.	Intervention cafeteria food improved significantly in all Mediterranean index categories except nuts, satisfaction improved significantly, however, diner’s consumption habits were not changed significantly when compared with the control community.
Seguin-Fowler 2020 [60]	Rural populations in the United States experience higher rates of obesity and are less likely to engage in health-promoting behaviors (e.g., adequate diet, physical activity) than urban populations	Environmental constraints in rural areas, including limited access to nutritious foods and opportunities for physical activity	Town 2- Wisconsin: population of approximately 2200 people; 9% of residents live in poverty - sought to change food environment at local middle school Town 3- Alaska: population of approximately 4700 people; 13% of residents live in poverty - sought to provide healthy food for students in Transition (homeless youth, couch-surfing or living in cars)”	• Point-of-purchase (town 2) • Increase availability (town 3)	*Point-of-purchase* - Changing cafeteria layout to encourage healthy food choices *Increase availability of healthy food* - Construct tiny food pantries to provide healthy foods for students in need	Limited description of results, feedback from leader’s interviews regarding challenges discussed.
Stluka 2019 [61]	High obesity prevalence and high percentage of Supplemental Nutrition Assistance Program (SNAP) participants in the counties	Not described	13 gardens	• Increase availability • Increase access	*Increase access to and availability of healthy foods* - Set up gardens in community to grow foods	Community garden was able to produce substantial amount of food. Some produce was donated to volunteers and food pantries. No mention of gardening effects on food security and diet quality.
Toft 2018 [63], Mikkelsen 2018 [64], Buch-Andersen 2021 [65], Toft 2023 [67], Toft 2017 [62] & Pedersen 2023 [66]	Project Sol	High proportion of citizens with a low socioeconomic position and high prevalence of health risk factors and non-communicable diseases	Multiple settings including schools, childcare centers, and supermarkets, local mass media and social media.	• Increase availability •Increase access • Point-of-sale • Promotion • Subsidies	*Increase access to and availability of healthy foods* Healthy snacks at childcare centers Breakfast club at schools *Point-of-sale* -Space management to promote fruit and vegetable sales - Co-collation of food items in a healthy meal - Candy-free checkouts *Promotion* -Activities in supermarkets (e.g. healthy treasure hunts/recipes), schools and childcare centers (e.g. taste activities/cooking events) -Promotion via mass media and social media (e.g. health recipes) *Subsidies* -20% discount on fruit and vegetables in 1 large supermarket for 3 months.	Qualitative interviews revealed positive remarks from community members about Project Sol, including components sustained/developed after project completion e.g. School herb and vegetable garden, school hen house [64]. Discount on fruit and vegetables increased sales of fruit and vegetables by 15.3% (*p* = 0.01). Space management only did not increase sales of fruit and vegetables [60]. Increase in sales of healthy foods with multi-component and mass media interventions [65]. No favorable effects of the intervention on BMI z-scores and WC in children [63].
Whelan 2023 [68]	Community food environment audit suggested that it was difficult to access healthy food in this area	A community food environment with an abundance of high-fat takeaway foods, where access to healthy food was poor and healthy options were usually more expensive.	YarriYak cafe is located within the premises of the Rural North West Health Service.	• Point-of-purchase	Traffic light food classification system according to the Victorian Healthy Choices Classification Guide identifies ‘green’ as the best choice, ‘amber’ as those to choose carefully and ‘red’ as those to be limited. The health policy requires 50% ‘green’ foods and no more than 20% ‘red’ foods/beverages.	Cafe displayed food and beverages as 68% green, 12% amber and 20% red. Average green sales were 77% of total sales, amber sales were 16% of total sales, red sales were 7% total sales. 26% of the customers used the ‘traffic light’ system to inform purchase all or most of the time.

Abbreviations:

PIU – Plate it Up

JCMS – Jefferson County Middle School

CVD – Cardiovascular Disease

SBP – School Breakfast Program

## Discussion

This is the first review to synthesize the international evidence on community involvement in rural food environment interventions. Studies covered multifaceted interventions in various environments where people procure and consume foods, from restaurants to community gardens. Community involvement was variable. Some rural communities were more highly involved in some strategies, such as increasing access and availability of food, but few details were provided in other strategies such as point-of-purchase interventions. It was difficult to determine whether there was an association between community engagement and positive primary outcomes of the program due to ambiguity in the details of community involvement reported. The focus of included papers primarily described intervention outcomes rather providing the specific methodology of community engagement. This means that there is little evidence globally to support best practice recommendations in involving rural communities in food environment interventions that target them.

The food environment is complex and constantly evolving. Several socio-ecological models of the food environment have been proposed to understand the interplay between the food environment and food consumption patterns of individuals [2, 69]. Evidence has suggested that healthier food environments result in higher diet quality amongst individuals [70]. As such, one of the first steps for researchers may be to evaluate the current food environment and identify areas where modifications may be required. However, an Australian review highlighted the absence of a standardized, reliable, sensitive and valid measurement tool to evaluate the rural food environment and inform appropriate local interventions [71]. This review also underscored the idea that each food environment is unique in its own way, thus driving the need for targeted measurement approaches [71]. The involvement of community stakeholders in the assessment process and fostering close partnership with researchers are crucial to identifying barriers and facilitators for successful food environment interventions [13] yet in this review, it was poorly reported. It must be noted that in one complex intervention where community involvement over a three-year period is well described, it took the form of several manuscripts [66–71]. 

The inclusion of community in promoting health is stipulated by the World Health Organization as a method of empowering people to control their own health [72]. Agency is a key component of empowering communities, with the Food and Agriculture Organization defining agency as “*the capacity for individuals or groups to make their own decisions about what foods they eat… and how that food is distributed within food systems*” [73]. Community based participatory research [74], co-creation and co-design [75] enable the inclusion of agency, community voices and lived experience from within a given context as part of development processes, while providing a structured research methodology. Inclusion of community has additional benefits, with a 2021 scoping review indicating transparency, more efficient policies and services, and an effective method of redistributing power as part of their findings in relation to health promotion [76]. Also, a 2021 meta-analysis reported moderate positive effects on community function outcomes when community was included, and small positive effects for health-promoting behaviour outcomes [77]. However, this same meta-analysis of co-creation in health research in 26 primary studies reported that co-creation did not extend through to the evaluation and dissemination stages [77]. 

Community involvement requires researchers to work collaboratively with retailers, community members and other interested parties to establish common objectives that facilitate the design and implementation of health-enabling food environment initiatives [78]. However, engaging with the community requires fostering relationships, developing close partnerships, and requires the sharing of time, resources, flexibility, decision-making and process orientation to accommodate the varied needs [77, 79]. Despite this, collaborative work is especially important when it comes to marginalized population groups [16]. Marginalized populations are often prevalent in rural areas, where their needs are often overlooked due to geographic isolation, resulting in reduced healthy food availability and access [7, 8]. The findings of our review identified that the community was involved to differing extents, but it was unknown if the engagement method was suitable in these programs and whether that has resulted in a positive food environmental change. A 2023 review suggested that there is a lack of standardized criteria for selecting suitable engagement methods in place [13]. The challenge instead is how to report community engagement strategies so that they can be assessed for suitability and/or adapted for replication in other health contexts or communities, which extends to the food environment.

A review by Slattery et al. (2020) has implied that co-design in research appears to be widely used but seldom described or evaluated in detail [80]. This aligns with our findings where the majority of reviewed papers have documented the importance of community involvement [42, 44, 47, 48, 54–58, 60–65] but details about the local contributors and the roles they played in the interventions were limited and ambiguous. A recent review by Vargas et al. (2022) on the co-creation of healthier food environments found similar results where it stated that insufficient details limits the evaluation of co-creation process effectiveness [16]. Evidence has suggested that realizing the potential of research co-design may require the development of clearer and more uniform terminology, better reporting of the activities involved and better evaluation [80]. A 2015 review by MacQueen et al. on evaluating community engagement in global health research also suggested the need to improve the evaluation of community engagement practices to foster an evidence-based understanding of effective strategies and facilitate the establishment of replicable best practices across research [81]. 

Few frameworks have been proposed over the years to evaluate program interventions and community involvement. The RE-AIM model, proposed in 1999, provides a comprehensive framework for understanding the real-world impact of programs, community based multi-level interventions and initiatives by focusing on five key elements: Reach, Effectiveness, Adoption, Implementation, and Maintenance [82, 83]. The PRODUCES framework (PRoblem, Objective, Design, (end-)Users, Co-creators, Evaluation, Scalability), described in 2019, proposed recommendations and a checklist for researchers to report on community involvement initiatives more broadly, aiming for reporting consistency and clearer insights on community involvement details [84]. However, based on our findings, it is not clear on how this can best be done for rural communities [77]. 

Strengths and limitations:

This review addresses a gap in the current rural populations’ literature, demonstrating a comprehensive overview of the use of community involvement in food environment modifications. However, it must be noted that grey literature, such as government and project reports, were not included in this review. Some food environment programs may have been missed due to not being published in an indexed journal and studies that did not explicitly state rurality or community involvement in the title or abstract might have been excluded in the screening stage. Additionally, data extraction and interpretation were challenging due to the heterogeneity of included studies and the lack of clear framework for data to be reported against, resulting in some data that was difficult to classify during the data extraction stage. Additionally, reporting interventions performed with First Nations populations separately may have reduced the number of insights and strategies found here.

## Conclusions

Future research is warranted to provide deeper insights into food environment research and to understand evidence-informed, best approaches to community engagement. To advance community involvement as an innovative collaborative approach in rural food environment modifications, consistent use of recognized and validated methodologies and frameworks for the food environment is needed to ensure the consistency of reporting across different studies. This will assist in developing a better understanding of the processes undertaken in engaging local communities, their effects on rural food environments, and how the research meets the needs of the community involved. Researchers have a responsibility to engage in systematic and authentic approaches to community involvement, including the fostering of relationships and partnerships with the sharing of resources and decision-making. With local community members co-creating context-specific interventions, rural health researchers will support the development of equitable food systems and enhance the evidence provided by interventions.

## Key References

- Taffere GR, Abebe HT, Zerihun Z, et al. Systematic review of community engagement approach in research: describing partnership approaches, challenges and benefits. Journal of Public Health-Heidelberg 2024; 32:185–205; 10.1007/s10389-022-01799-9.
  - ○This systematic review synthesised the approaches that had been reported to engage community in health research. They found a divergent methods and recognised a lack of standardised criteria for reporting engagement.
- Williams T, Thompson K, Brown C, et al. Assessing community readiness to reduce consumption of sugary drinks in remote Aboriginal and Torres Strait Islander communities: A useful tool for evaluation and co-design. Health Promot J Austr 2023; 34:30–40; 10.1002/hpja.639.
  - ○This manuscript describes the adaptation of the Community Readiness Tool, an existing framework that assesses community readiness for change. Specifically, it describes the process of co-designing the existing framework to be culturally appropriate for specific First Nations populations in remote areas of Australia.
- Karanja A, Ickowitz A, Stadlmayr B, et al. Understanding drivers of food choice in low- and middle-income countries: A systematic mapping study. Global Food Security-Agriculture Policy Economics and Environment 2022; 32:1615; https://doi.org/10061510.1016/j.gfs.2022.100615.
  - ○This systematic review maps sources of evidence of food choices for low and middle income countries. It describes a focus in the literature on urban-based food environments, with significantly less attributed to rural areas.

## Electronic Supplementary Material

Below is the link to the electronic supplementary material.

Supplementary Material 1

## Data Availability

No datasets were generated or analysed during the current study.
