# Supplementary material for: Understanding the Use of Community Involvement in Rural Food Environment Modifications: A Systematic Review
Source: Curr Nutr Rep. 2025 Jun 28;14(1):87. doi: 10.1007/s13668-025-00674-9 (PMC12206173; doi:10.1007/s13668-025-00674-9)
Supplement: Supplementary file 1 — Supplementary Material 1 [file 13668_2025_674_MOESM1_ESM.docx]

Supplementary material 1: Terms included in the search strategy

| # | Search terms |
| --- | --- |
| 1  Context | "Food outlet*" OR food OR "Food environment" OR "food supply" OR cafe* OR retail* OR restaurant* OR "recreation facility" OR supermarket* OR market* OR store* OR “health service*” OR “built environment” |
| 2  Exposure or intervention | (Supply adj2 distribution) OR (accessibility adj3 food) OR (availability adj3 food) OR (food adj2 choice) OR (food adj2 product) OR promotion* OR "choice behav*" OR Marketing/ or marketing OR price* OR placement OR incentive* OR “health promotion*” OR afford* |
| 3  Participants | Rural Population/ or "Rural Population" OR Remote OR "Regional Farming Community" OR "regional area*" OR rural OR "regional community" OR “non-urban” |
| 4  Condition or domain | Co-design OR co-creation OR co-production OR co-development OR community-led OR “Community Participation” OR “Community-Based Participatory Research” OR “community engagement” OR “Community-Institutional Relations” OR "Referral and Consultation” OR “community consultation” OR “civic engagement” OR stakeholder* OR “community intervention*” |
| 5 | 1 AND 2 AND 3 AND 4 |
